# Patients with langerhans cell histiocytosis and hypothalamic-pituitary involvement: insights from the HEROS study cohort

**DOI:** 10.1007/s11102-025-01576-3

**Published:** 2025-09-26

**Authors:** Hiba Masri Iraqi, Marina Tsoli, Mattia Barbot, Annamaria Colao, Diego Ferone, Miklos Toth, Ekaterina Pigarova, Amit Akirov, Lior Baraf, Yona Greenman, Mirjana Doknic, Gregory Kaltsas, Ilan Shimon

**Affiliations:** 1 Rabin Medical Center, Petah Tiqva, Israel; 2 Gray Faculty of Medical & Health Sciences, Tel Aviv University, Tel Aviv, Israel; 3 Laiko University Hospital, Athens, Greece; 4 Federico II University of Naples, Naples, Italy; 5 IRCCS Ospedale Policlinico San Martino, University of Genova, Genova, Italy; 6 Department of Internal Medicine and Oncology, Semmelweis University, Budapest, Hungary; 7 Soroka University Medical Center, Beer Sheva, Israel; 8 Endocrinology Research Centre, Moscow, Russia; 9 Tel Aviv-Sourasky Medical Center, Tel Aviv, Israel; 10 Neuroendocrine Department, Faculty of Medicine, University of Belgrade, Belgrade, Serbia; 11 Institute of Endocrinology, Rabin Medical Center- Beilinson Hospital, Petach Tikva, 49100 Israel; 12 Department of Medicine-DIMED, University of Padua, Padua, Italy; 13 Endocrinology Unit, University Hospital of Padua, Padua, Italy

**Keywords:** Langerhans cell histiocytosis, Pituitary, Hormones

## Abstract

**Purpose:**

Langerhans cell histiocytosis (LCH) is a rare disease involving multiple organs, including the endocrine system. This multicenter study aimed to characterize patients with hypothalamic- pituitary involvement in LCH.

**Methods:**

The Hypopituitarism European NeuroEndocrine Association (ENEA) Rare Etiologies Observational Study (HEROS) platform invited ENEA members to include patients with rare pituitary diseases like LCH. Demographic data, presenting symptoms, hormonal profile, imaging tests, treatment, and prognosis were retrieved.

**Results:**

Forty-eight patients (58% males) were included. Age at diagnosis was 22 ± 16.1 years, with 58% diagnosed as adults (> 18 years). The mean follow-up was 15.8 ± 10.6 years, 46% of the patients initially presented with bone lesions, 42% with lung involvement, and five were incidentally diagnosed. At diagnosis, 69% of the patients had arginine vasopressin deficiency (AVD), 42% had central hypogonadism, 25% hypothyroidism, and 12.5% hypocortisolism. Magnetic resonance imaging (MRI) was available in 41 patients, 73% of whom had pathology of the posterior pituitary/pituitary stalk. Visual disturbances were reported in only one patient. Diagnosis was histopathologically confirmed in all patients, mainly from extra-pituitary lesions. Transcranial biopsy was performed in five patients, and two underwent transsphenoidal intervention. During follow-up, 27% of the patients developed new AVD and five acquired new anterior pituitary hormone deficiency. There was no disease-related mortality during follow-up.

**Conclusions:**

Patients with LCH and hypothalamic-pituitary involvement remained clinically stable during long-term follow-up. However, new hormonal deficits may develop years after diagnosis, with most patients ultimately experiencing AVD.

## Introduction

Langerhans cell histiocytosis (LCH) is a disease characterized by the proliferation of cells of the mononuclear phagocyte system [1]. The pathogenesis of LCH is not fully understood, exhibiting varying patterns of clinical involvement and outcomes [2, 3]. Incidence of the disease varies according to age, with a higher prevalence in children, reaching 3–5 cases per million per year, compared to1-2 cases per million among adults [4–6].

Many organs are susceptible to LCH infiltrates; the disease may manifest as localized lesions in specific organs or as a disseminated disease involving multiple organs [2, 7]. Commonly affected sites include bones, lungs and skin, which are involved in up to 50% of cases. The endocrine system, particularly the hypothalamic-pituitary axis, is also frequently affected. While the neurohypophysis is involved in up to 25% of cases, anterior pituitary deficiencies are also prevalent in 20% of patients with LCH [7–9]. This involvement is believed to result from clonal proliferation in the pituitary region and an inflammatory response of the local immune system, leading to cytokine storm, contributing to the destruction of the pituitary cells [10].

The diagnosis of LCH relies mainly on the histopathological findings in biopsies from suspected lesions, which are performed whenever feasible. In cases where a biopsy is not possible, diagnosis may rely on radiological and clinical features. Patients diagnosed with LCH should undergo a comprehensive work-up, including endocrine evaluation to confirm or exclude hypothalamic-pituitary involvement [7].

This study aimed to assess the spectrum of hypothalamic-pituitary dysfunction in patients with LCH, characterize the clinical features of these patients, and evaluate their long-term outcomes during follow-up in a large cohort of LCH patients with neuroendocrine involvement.

## Methods

The Hypopituitarism ENEA Rare Etiologies Observational Study (HEROS) platform is an electronic survey introduced by the European NeuroEndocrine Association (ENEA) in 2016 for ENEA members to include patients with rare pituitary diseases- LCH, Idiopathic arginine vasopressin deficiency (AVD), Hypophysitis (lymphocytic & others) and Sarcoidosis.

Patients with endocrinopathies and LCH were recruited from 9 pituitary centers in 6 countries- Greece, Hungary, Israel, Serbia, Italy, and Russia. Diagnosis of LCH in suspected patients was based on biopsies usually from organs involved other than the pituitary. This retrospective study collected demographic data, age at diagnosis, organs involved, autoimmunity background, clinical symptoms at presentation, pituitary imaging, visual impairment, anterior pituitary dysfunction, AVD, glucocorticoid treatment, systemic treatments, and mortality. In 2023, investigators were contacted to report the updated status of hormonal deficiencies of the anterior and posterior pituitary gland, MRI results, and mortality.

The Institutional Review Board approved the study at each participating center, in accordance with local regulatory requirements.

## Statistical analysis

Continuous parameters were summarized by mean and standard deviation (SD), and categorical parameters by proportion. Groups were compared for continuous parameters using the t-test and Mann-Whitney test, and for categorical parameters using the chi-square test. A two-sided *P* value of < 0.05 was considered significant.

## Results

The study cohort included 48 patients (28 males 58%) (Table 1) from several countries, including 18 from Greece, 12 from Italy, 8 from Israel, 5 from Serbia, 4 from Hungary, and one from Russia. The mean age at diagnosis was 22 ± 16.1 years (median, 20 years; range, 1–56 years), with 58% (28) of patients diagnosed in adulthood (> 18 years). Males were diagnosed at a mean age of 19.5 ± 14.5 years, while females were diagnosed at an older age of 25.5 ± 17.5 years (*p* = 0.2). The mean follow-up duration was 15.8 ± 10.6 years.

**Table 1:** A cohort of patients diagnosed with LCH

	**N=48**
Male	28 (58%)
Age at mean (yrs)	22±16.1
Follow up, mean+SD (yrs)	15.8±10.6
Presentation at diagnosis	
Bone	22 (46%)
Lung	20 (42%)
Incidental	5 (10%)
Fever	3 (6%)
Pituitary hormone deficiency at presentation	
Hypogonadism	20 (42%)
Central Hypothyroidism	12 (25%)
Hypocortisolism	6 (12%)
AVD	33 (69%)
GH deficiency^¶^	5 (12%)
AVD as presenting symptom	20 (42%)
Imaging	**N=41**
Posterior pituitary/stalk abnormality	30 (73%)
Anterior pituitary mass	4 (10%)
Suprasellar mass	8 (20%)

¶ Out of 41 patients with reported GH status

### Presenting features

The most common presenting symptoms were related to bone disease in 46% of patients and lung disease in 42% of patients. Additionally, three patients were diagnosed during the evaluation of fever, five through incidental findings, and 42% of patients had AVD as a presenting symptom (Table 1). In nine patients presented with AVD, initial workup revealed anterior pituitary hormone deficiency. In two cases thyroid involvement was reported without more information on cytologic findings.

### Endocrine dysfunction

Anterior pituitary hormone deficiencies were identified in 48% of patients; 42% had hypogonadotrophic hypogonadism, 25% central hypothyroidism, 12% presented with central hypocortisolism, and 69% of patients had AVD at diagnosis. Five patients had three hormone deficits, five had two hormones affected, and 13 presented with a single hormone deficit.

### Pituitary imaging

Magnetic resonance imaging (MRI) at diagnosis was available for 41 patients (Table 1). Of these, 30 (73%) exhibited posterior pituitary and/or pituitary stalk abnormality, with 23 (76%) of these patients having AVD. Six patients without posterior pituitary and/or pituitary stalk abnormality also had AVD. An anterior pituitary mass was observed in four patients, eight patients had suprasellar masses. Four patients had a normal sellar MRI. Visual field abnormality was evident in only one patient.

### Histopathological findings

The diagnosis of LCH was confirmed by biopsies. In 32 cases a biopsy was obtained from extra pituitary lesions, five patients underwent trans-cranial biopsy and two patients had trans-sphenoidal biopsy of the pituitary. In nine patients the site of biopsy was not available.

### Treatment and outcomes

Therapies included glucocorticoids, radiotherapy, and chemotherapy. Among eight patients treated with pharmacological doses of glucocorticoids, five achieved remission and three showed symptom improvement. Two of these patients had partial hormonal recovery: AVD improved in one, and thyroid dysfunction in another.

Seven patients underwent radiotherapy for their lesions, leading to symptom improvement in three cases. None of the patients received sellar irradiation.

Various chemotherapeutic medications were administered to 23 patients, either alone or in combination (Cladribine, Vinblastine, Adriamycin, Vincristine, Etoposide,6-mercaptopurin, Azathioprine, Methotrexate, and Cyclophosphamide). In 18 patients, vinca agents (Vinblastine, Vincristine) were used; five patients were treated with a single drug without vinca agents (three treated with cladribine, one with azathioprine, and one with etoposide). Chemotherapy resulted in permanent remission in two patients, two-year remission in four patients, and symptom improvement in ten. Hypogonadism resolved in one patient following chemotherapy.

Combination therapy with glucocorticoids, radiotherapy, and chemotherapy was administered to one patient and radiotherapy with chemotherapy to another.

### Long-Term outcomes

At the last follow-up visit, 46 patients had AVD deficiency (96% of patients). In 13 patients AVD developed during a mean follow-up of 11.8 ± 10.6 years since LCH diagnosis (median, 5.6 years; range, 0.25-36 years).

Five patients without prior anterior pituitary dysfunction developed at least one new hormone deficiency, resulting in 28 patients having anterior pituitary deficiency at end of follow-up; 50% had hypogonadism, 46% central hypothyroidism and 23% hypocortisolism (Table 2). All patients with hypothyroidism or hypocortisolism received replacement therapy. Among those with hypogonadism, 12 males were treated with testosterone and 5 females were given estrogen replacement. Growth hormone replacement was given to six of 12 patients with GH deficiency.

**Table 2:** Pituitary hormones at baseline and end of follow-up

	Baseline	Resolved	Added	End of follow-up
AVD	33 (69%)	0	13	46 (96%)
Hypogonadism	20 (42%)	1	5	24 (50%)
Hypothyroidism	12 (25%)	0	10	22 (46%)
Hypocortisolism	6 (12%)	0	5	11 (23%)
GH deficiency^¶^	5 (12%)	0	7	12 (29%)

¶Out of 41 patients with reported GH status

Among 20 patients who had DI as a presenting symptom, systemic involvement was revealed in 13 during workup and follow-up; 6 had bone lesions, 5 had lung involvement and two developed both bone and lung lesions.

### Age-Specific characteristics

Twenty patients were diagnosed in childhood (mean age of 6.2 ± 5 years; median, 5 years), and 28 patients were diagnosed as adults (mean age of 33.3 ± 11.2 years; median, 34 years). Bone lesions were more common at diagnosis in younger patients (12 children vs. 9 adults, *p* = 0.05), while lung involvement was observed in 6 children and 14 adults (*p* = 0.2). AVD was present at diagnosis in 75% of pediatric patients and 64% of adults and was the presenting symptom in 45% and 35%, respectively (Table 3).

**Table 3:** Patients diagnosed with LCH by the age at diagnosis

	Young (≤ 18) *N* = 20	Adult(> 18) *N* = 28	*P* value
Age at diagnosis	6.2 ± 5	33.3 ± 11.2	
Male	13(65%)	15(54%)	NS
Presentation at diagnosis			
Bone	12(60%)	9(32%)	0.05
Lung	6(30%)	14(50%)	NS
Incidental	2(10%)	3(11%)	NS
Fever	2(10%)	1(4%)	NS
Pituitary hormone deficiency at presentation			
Hypogonadism	7(35%)	14(50%)	NS
GH deficiency	3(15%)	2 (7%)	NS
Hypothyroidism central	7(35%)	4(14%)	NS
Hypocortisolism	3(15%)	3(11%)	NS
AVD	15(75%)	18(64%)	NS

## Discussion

In this multicenter study of patients with LCH followed in European pituitary centers, we demonstrate the heterogeneity of pituitary involvement among young and adult patients, with a large number of adult patients included in addition to patients diagnosed during childhood. During prolonged follow-up, almost all patients demonstrated posterior pituitary involvement.

LCH is a rare disease and epidemiological studies reveal different incidence rate across various countries, in Europe reported incidence among children is between 2.24 and 8.9 per million but with different study design [11–15], in the United States one study reported the SEER data with incidence of < 0.7 per million reporting disseminated disease [16]. One study from Greece with adult patients reported incidence of 1.58 per million [17]. Reports from the United States indicate ethnic disparities in disease incidence, with lower rates among Black populations relative to White populations, and higher rates among Hispanic compared to non-Hispanic individuals [16, 18]. In contrast, our study population was primarily European, where a previous study from England has not identified such ethnic differences, though non-White groups are still underrepresented [15].

The age at LCH diagnosis in this cohort corresponds with the previously reported two peak pattern. Earlier studies have reported one peak at 1–4 years of age in children [13, 19, 20]. In the young patient’s group age was similar to the report by Alexander et al. of 141 pediatric patients with LCH [21]. Among adults with LCH, the mean age at diagnosis was consistent with the reported mean age in the fourth decade for adults with LCH [6]. Male predominance was described in early reports reaching a 2:1 ratio [22], and a ratio of 1.8:1 was reported in 121 pediatric patients diagnosed with LCH [19]. In our cohort, there was a male predominance of 1.4:1 in the whole cohort, reaching a ratio of 1.8:1 in children. This suggests that there may be a variance in dominance based on the age at diagnosis. However, a study conducted with childhood LCH patients in France reported a ratio of 1.2:1 [21] with the same ratio reported also in a large adult cohort with LCH patients [9].

The extra-pituitary organs affected in our study were bones and lungs in 46% and 42% of patients, respectively. In a large series of patients with LCH, bones were more often affected than lungs, 57% and 40%, respectively [9]. Bone involvement is prominent in up to 80% of children with LCH [23], and in the report by Makras et al. bone and lung involvement were similar also being present in 75% of a small series of 17 adult patients [24]. In the analysis of young and adult patients in our cohort, bone involvement was significantly more prevalent in children than in adults (Table 3). These differences in the reported organ involvement reflect the difference in the age of the study population. Studies reporting predominantly children participants reported prominent bone involvement.

The pituitary gland is vulnerable, frequently involved in LCH, mainly the posterior pituitary lobe. Endocrine manifestations are reported in 17–28% of patients in LCH cohorts with varying follow-up time of 5–11 years [21, 25–27]. Our study was designed to investigate patients referring to endocrinology clinics with 48% of patients exhibiting at least one anterior pituitary hormone affected at diagnosis, which rose to 58% at the last follow-up visit. Alexander et al. reported a similar proportion to our study having also included children with pubertal delay [21]. In previous studies, growth hormone (GH) deficiency and hypogonadism were the leading pituitary hormonal axes affected (16–19). In our research, hypogonadism was more frequent than GH deficiency, reaching 50% of the patients at the last follow-up compared to 29%, respectively, and may be related to the under-diagnosis of GH deficiency and the study’s design.

Hormonal replacement therapy is given to patients with anterior pituitary deficiencies. While there are concerns about the impact of GH replacement therapy on LCH activity, previous studies have reported no significant adverse effects on LCH lesion recurrence in both children and adults across large patient groups [13, 20]. In this study six patients received GH replacement, and data were not available regarding adverse effects other than mortality.

Affecting 12–30% of all Langerhans Cell Histiocytosis (LCH) patients, AVD is a relatively common manifestation of this condition. Cohort studies indicate that 88–100% of LCH patients monitored in endocrine departments report AVD (Table 4). In our study, 96% of patients ultimately developed AVD; 69% of patients presented with AVD at the time of diagnosis compared with 41% in the series by Makras et al. [24]. In 42% of our patients, AVD was the presenting symptom compared to 33–46% in other series (Table 4). In our cohort, 27% of the patients developed new AVD during a mean follow-up time of 11.8 years and up to 36 years after LCH diagnosis, emphasizing the importance of long-term clinical follow-up for these patients. Moreover, new anterior pituitary deficiencies developed years after the initial diagnosis, resulting in 58% of all cohort having at least one anterior pituitary hormone deficiency; other studies also described a prevalence of 58–67% [28, 29].

**Table 4:** Studies investigated hypothalamic-pituitary involvement in LCH patients, AVD prevalence at diagnosis and development during follow-up

	*N*	Age	Adults	Children	Follow-up years	AVD	AVD at presentation	Time to new AVD disgnosis, yrs
Current	48	22 ± 16.1	28 (58%)	20 (42%)	15.8 ± 10.6	46 (96%)	33 (69%)	12.3 ± 11; Range 0.25-36
Kaltsas et al.^−20^	12	Median 28.5, (range 1–47)	8 (67%)	4 (33%)	Median 11.5; Range 3–28	12 (100%)	4 (33%)	Median 2; range 0–20
Markas P. et al.^−15^	17	median 21.5 (range 3–57)	17		NA	16 (94%)	7 (41%)	NA
Alexander A et al.^12 ¶^	24	NA		24 (100%)	NA	21 (88%)	11(46%)	Mean 3; range 1–8

¶ Data relates to patients with endocrinopathies from the whole cohort of 141 patients with LCH

In our cohort, 76% of patients with initial imaging information exhibited abnormalities of the posterior pituitary and infundibulum at presentation. Among patients with AVD at presentation, only 73% had abnormalities on imaging, while the previously reported cohorts described the absence of the posterior pituitary bright spot in all patients [24, 30]. Interestingly, seven patients with abnormal imaging of the posterior lobe or the infundibulum did not have AVD at diagnosis. However, six of them eventually developed this functional disturbance, emphasizing the role of pituitary imaging in LCH patients.

Systemic treatments administered to patients with LCH usually have mild beneficial effects on endocrine dysfunction, and pituitary damage usually does not improve [7, 9, 23]. In our cohort, treatments also led to a minimal effect; hypogonadism resolved only in one patient.

Our study has several limitations. First, it is retrospective in design and uses electronic data recording. Second, there was no standardization of hormonal deficiency diagnosis, imaging workup for extrapituitary involvement, follow-up, and some of the patients included did not have complete data. The electronic sheet did not contain information on all potential extrapituitary sites or specify the bone lesions and their locations. However, it reflects the clinical characteristics and course of the disease in the context of hypothalamic-pituitary axis involvement.

In conclusion, patients with LCH with pituitary endocrine manifestations preceding diagnosis or when presented need long-term follow-up to identify new endocrine disturbances, with almost all patients developing AVD. Whether this should be implicated in all patients with LCH, even without initially hormonal deficits, needs more investigation.

## Data Availability

No datasets were generated or analysed during the current study.
