# Correction: Patients with Langerhans cell histiocytosis and hypothalamic-pituitary involvement: insights from the HEROS study cohort

**DOI:** 10.1007/s11102-025-01631-z

**Published:** 2026-03-25

**Authors:** Hiba Masri Iraqi, Marina Tsoli, Mattia Barbot, Annamaria Colao, Diego Ferone, Miklos Toth, Ekaterina Pigarova, Amit Akirov, Lior Baraf, Yona Greenman, Mirjana Doknic, Gregory Kaltsas, Ilan Shimon

**Affiliations:** 1 Rabin Medical Center, Petah Tiqva, Israel; 2 Gray Faculty of Medical &Health Sciences, Tel Aviv University, Tel Aviv, Israel; 3 Laiko University Hospital, Athens, Greece; 4 Federico II University of Naples, Naples, Italy; 5 IRCCS Ospedale Policlinico San Martino, University of Genova, Genova, Italy; 6 Department of Internal Medicine and Oncology, Semmelweis University, Budapest, Hungary; 7 Soroka University Medical Center, Beer Sheva, Israel; 8 Endocrinology Research Centre, Moscow, Russia; 9 Tel Aviv-Sourasky Medical Center, Tel Aviv, Israel; 10 Neuroendocrine Department, Faculty of Medicine, University of Belgrade, Belgrade, Serbia; 11 Institute of Endocrinology, Rabin Medical Center- Beilinson Hospital, Petach Tikva, 49100 Israel; 12 Department of Medicine-DIMED, University of Padua, Padua, Italy; 13 Endocrinology Unit, University Hospital of Padua, Padua, Italy

**Correction: Pituitary (2025) 28:104**

**https://doi.org/10.1007/s11102-025-01576-3**

In this article, the author Dr. Mattia Barbot was missing from the author group and the author is affiliated to “Department of Medicine-DIMED, University of Padua, Padua, Italy” and Endocrinology Unit, University Hospital of Padua, Padua, Italy.

The original article has been corrected.

